# Functional Evidence of *CCDC186* as a New Disease-Associated Gene with Endocrine and Central Nervous System Alterations

**DOI:** 10.3390/ijms241512319

**Published:** 2023-08-01

**Authors:** Luisa Arrabal, Gerard Muñoz-Pujol, Inmaculada Medina Martínez, Laura Gort, Judit García-Villoria, Susana Roldán, Frederic Tort, Antonia Ribes

**Affiliations:** 1 Pediatric Neurology Department, Hospital Virgen de las Nieves, 18014 Granada, Spain; luisillaarrabal@hotmail.com (L.A.); inmamed@hotmail.es (I.M.M.); sroldanaparicio@gmail.com (S.R.); 2 Section of Inborn Errors of Metabolism-IBC, Department of Biochemistry and Molecular Genetics, Hospital Clínic Barcelona, IDIBAPS, CIBERER, 08028 Barcelona, Spain; gemunoz@recerca.clinic.cat (G.M.-P.); lgort@clinic.cat (L.G.); jugarcia@clinic.cat (J.G.-V.)

**Keywords:** CCDC186 mutations, seizures, hypomyelination, low levels of cortisol, low levels of insulin, growth hormone deficiency, non-ketotic hypoglycemia, dense-core vesicles, DCVs

## Abstract

CCDC186 protein is involved in the maturation of dense-core vesicles (DCVs) in the trans-Golgi network in neurons and endocrine cells. Mutations in genes involved in DCV regulation, other than *CCDC186*, have been described in patients with neurodevelopmental disorders. To date, only one patient, within a large sequencing study of 1000 cases, and a single case report with variants in *CCDC186*, had previously been described. However, no functional studies in any of these two cases had been performed. We identified three patients from two gypsy families, unrelated to each other, with mutations in the *CCDC186* gene. Clinically, all patients presented with seizures, frontotemporal atrophy, hypomyelination, recurrent infections, and endocrine disturbances such as severe non-ketotic hypoglycemia. Low levels of cortisol, insulin, or growth hormone could only be verified in one patient. All of them had a neonatal onset and died between 7 months and 4 years of age. Whole exome sequencing identified a homozygous variant in the *CCDC186* gene (c.2215C>T, p.Arg739Ter) in the index patients of both families. Protein expression studies demonstrated that CCDC186 was almost undetectable in fibroblasts and muscle tissue. These observations correlated with the transcriptomic analysis performed in fibroblasts in one of the patients, which showed a significant reduction of *CCDC186* mRNA levels. Our study provides functional evidence that mutations in this gene have a pathogenic effect on the protein and reinforces CCDC186 as a new disease-associated gene. In addition, mutations in *CCDC186* could explain the combined endocrine and neurologic alterations detected in our patients.

## 1. Introduction

**Figure 1:**
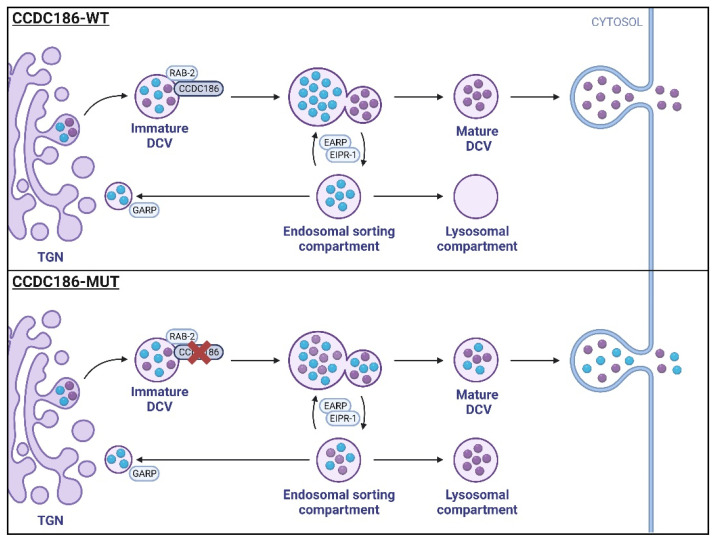
Schematic representation of dense-core vesicle (DCV) biogenesis and cargo sorting in CCDC186-WT (wild-type) and CCDC186-MUT (mutant) cells: (CCDC186-WT) Immature DCVs containing soluble cargo are formed at the trans-Golgi network (TGN). Cargo sorting is facilitated by the active, GTP-bound RAB-2 and CCDC186, among other factors. Maturation steps, including cargo acidification and processing, lead to the formation of mature DCVs, which are stored until stimulated release. Cargo not destined for secretion is transported to the endosomal sorting compartment via the endosome-associated recycling protein (EARP) complex and EARP inhibitor protein (EIPR-1). From the endosomal compartment, cargo can be shuttled back to the TGN via the Golgi-associated recycling protein (GARP) complex or processed for lysosomal degradation. CCDC186-MUT impairs cargo sorting in DCVs, resulting in the secretion of incorrectly sorted cargo. Furthermore, cargo may be misdirected towards lysosomal degradation, leading to reduced concentrations of secreted cargo.

The coiled-coil domain-containing (CCDC) proteins are implicated in many physiological and pathological processes, such as the interaction with molecular components of signaling pathways or their physiological functions at the cellular and organ levels, including cancer processes [1]. It is estimated that about 10% of an organism’s proteome contains coiled-coil-forming sequences [2]. Among them, CCDC186 is involved in secretory dense-core vesicles (DCVs) trafficking, which are specialized organelles of neurons and endocrine cells involved in the release of neuromodulators. DCV secretion regulates a wide variety of physiological processes, including neuronal development, synapses, and cell survival [3,4]. The biogenesis of DCVs is a complex and highly regulated process that requires the initial generation of granules at the trans-Golgi network, followed by several maturation steps and cargo sorting before secretion [3,5]. Although the molecular mechanisms are not completely understood, in recent years, several proteins (including CCDC186) have been reported to be involved in this complex and highly regulated process [4]. CCCP-1, the CCDC186 orthologue in *C. elegans*, was established as a downstream effector of RAB-2, one of the many Rab GTPases involved in DCV trafficking [3,4,6]. In addition, it colocalizes with the endosome-associated recycling protein (EARP) complex and the EARP interactor, EIPR-1, which are both responsible for sorting and recycling cargo through the endosomal compartment [7]. Additional studies performed in its worm ortholog have demonstrated a role for this protein as a DCVs trafficking regulator in neurons [5]. Later, an essential role of Otg1 (CCDC186 in humans) in vesicle trafficking, hormone secretion, metabolic regulation, and postnatal survival in mice was demonstrated [8]. Therefore, it is postulated that mutated CCDC186 impairs cargo sorting in DCVs, resulting in the secretion of incorrectly sorted cargo [3,4,5] (Figure 1).

The proper release of neuromodulators is essential for the appropriate function and development of the nervous system. In fact, patients with mutations in protein members of the EARP Complex and the Golgi-associated retrograde protein (GARP) have been reported to be associated with neurodevelopmental disorders. Among the five proteins associated with these complexes, only patients with mutations in three of them (VPS53, VPS51, and VPS50) have been reported [9,10,11,12,13]. VPS53 was the first to be associated with human disease and patients were characterized by severe early-onset neurodegeneration with profound intellectual disability, progressive microcephaly, spasticity, and early-onset epilepsy [9], or developmental delay, limb and facial edema, intractable epilepsy, optic atrophy, and dysmorphic features [10]. Concerning VPS51, only two families have been reported, and the clinical picture was characterized by severe global developmental delay, pontocerebellar abnormalities, microcephaly, hypotonia, epilepsy, and several systemic and peripheral dysfunctions [11,12]. Recently, two patients with mutations in VPS50 with neurodevelopmental disorder and neonatal cholestasis have also been described [13]. To date, *CCDC186* disease-associated variants have only been reported in one patient within a large sequencing study of 1000 cases from Saudi Arabia [14] and in a single patient presenting developmental delay, refractory epilepsy, and failure to thrive [15]. However, the association of CCDC186 deficiency with the clinical phenotype still remains unclear because of the lack of evidence about the impact of the identified variants on protein function, as well as the lack of additional clinically compatible patients that provide a comprehensive description of this new disease.

Here, we identified three additional patients from two unrelated families harboring a novel homozygous truncating mutation in the *CCDC186* gene. All of them presented a fatal course of the disease and displayed very homogeneous clinical phenotypes characterized by microcephaly, brain atrophy, refractory epilepsy, and endocrine alterations. In addition, we demonstrated a significant reduction of *CCDC186* mRNA and protein levels in patient samples, providing functional evidence of the pathogenic effect of *CCDC186* mutations on the encoded protein. Altogether, our observations reinforce *CCDC186* as a new disease-associated gene.

## 2. Results

### 2.1. Identification of CCDC186 Variant

**Figure 2:**
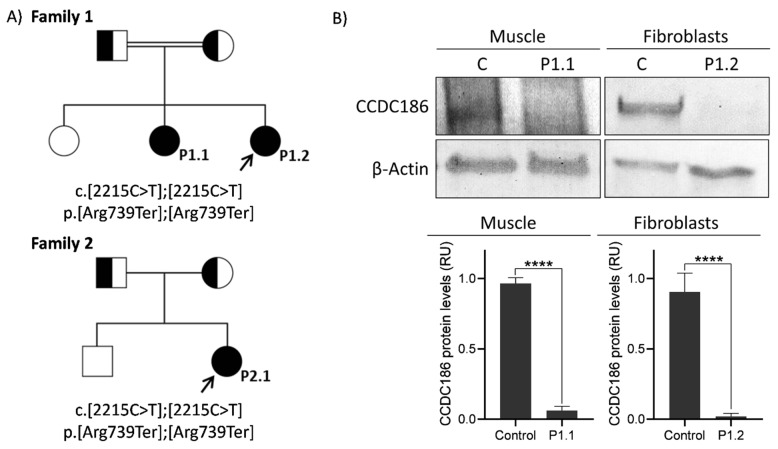
Pedigree and CCDC186 protein analysis. (**A**) Pedigrees showing three affected individuals from two unrelated families carrying the same homozygous mutation in *CCDC186* (c.2215C>T; p.Arg739Ter). Healthy parents were carriers. Arrows indicate the index case of each family. (**B**) Western blot analysis of CCDC186 in patients’ muscle tissue and fibroblasts showed undetectable levels of CCDC186 protein compared with control individuals. *β*-actin was used as a loading control. P, patient; C, control; RU, relative units. **** indicates *p* < 0.0001.

Whole exome sequencing (WES) analysis identified a homozygous variant (chr10-115887398G>A, c.2215C>T, rs767520724) in *CCDC186* (NM_018017.4) in the index cases of both families (Figure 2A). This substitution generates a premature termination codon predicted to truncate the CCDC186 protein at position 739 (p.Arg739Ter). Although the variant was present in the gnomAD database (accessed 2022), its frequency was extremely low, as it was only detected in one out of 250,968 alleles (0.0004%). Sanger sequencing confirmed the homozygous status of family 1 index case (P1.2), as well as of her affected sister (P1.1) in a DNA sample obtained from a small muscle biopsy, stored frozen for several years. The two families are unrelated, but they are both of gypsy origin from the same geographic area of Spain. Segregation studies confirmed the carrier status of the healthy parents in both families. More information about the position of the mutation and links to relevant databases can be found on the Franklin webpage (https://franklin.genoox.com/clinical-db/variant/snp/chr10-115887398-G-A?app=acmg-classification&popup=registration, accessed on 26 July 2023).

### 2.2. Analysis of CCDC186 Protein Expression

To determine the functional impact of the c.2215C>T variant, we analyzed the levels of CCDC186 protein in P1.1 muscle biopsy, as well as in P1.2 fibroblasts by Western blot. Results demonstrated the deleterious effect of the c.2215C>T substitution, as CCDC186 protein levels were almost undetectable in both affected siblings compared to control individuals (Figure 2B). No material was available from P2.1.

### 2.3. RNA Sequencing

**Figure 3:**
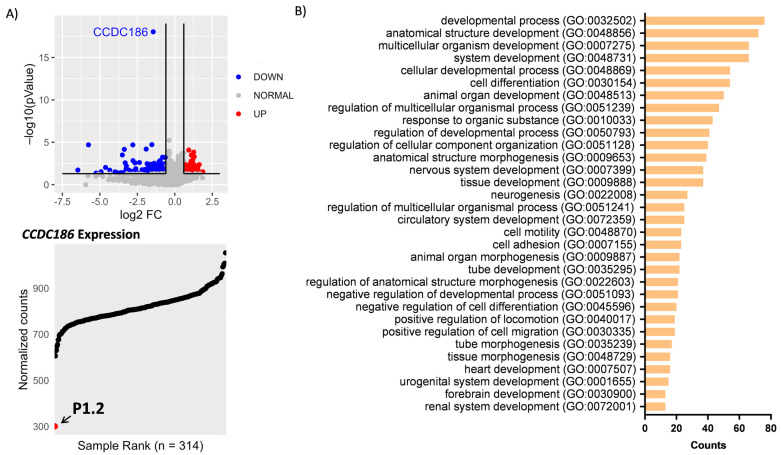
Transcriptomic analysis and differentially expressed genes. (**A**) RNA-seq analysis performed in P1.2 fibroblasts. RNA-seq data were analyzed using DROP, a statistical method based on a one versus all approach. Upper panel: Volcano plot showing gene-level significance (−log10 *p*-Value) against log2 FC. *CCDC186* (in blue) is the most downregulated gene in this individual. Lower panel: expression rank plot showing P1.2 (red dot) as the individual with the lowest levels of *CCDC186* expression among the entire cohort of transcriptomes (n = 314). (**B**) Statistical over-representation test of differentially expressed genes showed a significant enrichment, particularly for the processes involved in morphogenesis and development.

In addition to WES, RNA-seq was performed on P1.2 fibroblasts. Interestingly, analysis of the transcriptomic data also prioritized *CCDC186* as the candidate disease gene since its expression was significantly reduced. Moreover, *CCDC186* mRNA levels were the lowest of the entire cohort of transcriptomes analyzed (Figure 3A).

### 2.4. Functional Annotation of Differentially Expressed Genes

The availability of transcriptomic data of P1.2 prompted us to analyze whether the profile of differently expressed genes (DEG) could provide additional information about the physiopathology of CCDC186 deficiency. Therefore, we performed a statistical over-representation test on the significantly down and upregulated genes. Results showed enrichment in genes involved in morphogenesis and development (Figure 3B).

## 3. Discussion

*CCDC186* encodes a coiled-coil domain-containing protein that is involved in the maturation of dense-core vesicles (DCVs) at the trans-Golgi network in neurons and endocrine cells [3,4,5,8]. To date, variants in *CCDC186* have only been reported in two unrelated individuals presenting with failure to thrive, visual impairment, neurodevelopmental delay, hypotonia, and brain atrophy [14,15]. The patient reported by Brugger et al. [15] was clinically characterized in detail, whereas the other one was reported within a large cohort study, and the main phenotypic traits were only summarized in a table format [14]. Regardless of these descriptions, the association of *CCDC186* mutations with this pathologic condition has not been fully established, as functional studies have not been performed in any of the two individuals reported so far. Although in both cases, the identified variants are “nonsense”, they are considered “variants of uncertain significance”, and disease causality has not been completely proven.

Here we describe three additional patients from two unrelated gypsy families carrying a novel *CCDC186* variant in homozygosity (c.2215C>T, p.Arg739Ter) and provide the first functional evidence demonstrating the impact of a *CCDC186* variant on the encoded protein. The c.2215C>T variant was predicted to generate a premature termination codon. In fact, analysis of protein expression in the affected siblings of family 1 (Figure 2B) demonstrated the deleterious effect of this variant since CCDC186 protein was extremely reduced, almost undetectable in both patients. These observations correlated with the transcriptomic analysis performed in one of the siblings (P1.2), which showed a significant reduction of *CCDC186* mRNA compared to controls (Figure 3A), and clearly suggested that the mutated transcript may be subjected, at least in part, to nonsense-mediated mRNA decay. Altogether, our data demonstrated that the homozygous c.2215C>T variant could be pathogenic by dramatically reducing mRNA and protein levels in muscle and fibroblasts. Since the CCDC186 disease-associated variants described until now are nonsense mutations, the use of read-through enhancers could be considered as a potential therapy for this disease.

The siblings of family 1 were part of a consanguineous gypsy family. Therefore, the additional contribution of variants in other genes to the clinical phenotype could not be completely excluded. To consider this possibility, in addition to the nonsense *CCDC186* pathogenic mutation, we searched for variants located in homozygous stretches of P1.2. Our analysis identified rare “missense” variants in nine genes (Supplementary Materials Tables S1–S3). Notably, with the exception of *CCDC186*, none of them were prioritized. Similarly, the analysis performed on the previously reported CCDC186 patient [15], also from a consanguineous family, did not report any contribution of the additional identified variants to the clinical phenotype. These observations, together with the patient’s clinical overlap, reinforce *CCDC186* as the cause of the disease in these families.

Interestingly, all patients exhibited very uniform clinical symptoms. The main clinical, biochemical, and molecular data are summarized in Table 1. The Spanish individuals presented first symptoms during the neonatal period, while the patient reported by Brugger et al. [15] started symptoms at 4 months of age. However, all of them had a fatal course of the disease and died on or before the age of 4 years. The most prominent neurological features observed in all patients were microcephaly, developmental delay, muscular hypotonia, seizures, and MRI alterations such as frontotemporal atrophy and thin corpus callosum. Apnea episodes were only reported in our patients. Non-ketotic hypoglycemia was also one of the characteristic findings of our patients, but endocrine pancreas insufficiency was suspected in all of them. The previously reported patient [15] showed gastrointestinal problems, including exocrine pancreas insufficiency, which was also the case in one of the Spanish patients (P1.2) reported here. All these clinical symptoms are in agreement with the proposed function of the CCDC186 protein in DCV biogenesis. DCVs are secretory organelles of neuronal and endocrine cells that play a role in the regulation of processes such as synaptic plasticity, glucose homeostasis, and feeding habits [3,4,5,8]. Therefore, the impairment of DCV function due to CCDC186 deficiency could explain the characteristic combination of endocrine and central nervous system alterations observed in all patients, including the patient reported by Brugger et al. [15]. This hypothesis agrees with previous studies showing that *CCDC186* knock-out mice result in postnatal lethality, aberrant glucose homeostasis, and defective hormone secretion that leads them to hypoglycemia [8]. In this regard, it is interesting to remark that, among patients with other defective proteins of EARP/GARP complexes (VPS50, VPS51, or VPS53), the endocrine alterations are restricted to CCDC186 deficient patients (Table 1), while all of them share most of the neurological symptoms [9,10,11,12,13].

On the other hand, RNA-seq analysis of differentially expressed genes showed an over-representation of genes involved in processes associated with development. Although these data are consistent with developmental defects of patients, RNA-seq analysis of additional CCDC186 patients is desirable to confirm these observations and fully clarify the transcriptomic profile associated with CCDC186 deficiency in humans.

The study has certain limitations that should be acknowledged. Although our findings provide evidence of the pathogenicity of *CCDC186* mutations, as supported by the description of three additional patients, the sample size is still small. Another limitation of the present study is the lack of functional complementation studies in patient cells due to the lack of well-characterized biochemical or cellular phenotypes in CCDC186-deficient cells. Addressing these limitations through a larger series of CCDC186 patients would contribute to a deeper insight into CCDC186’s knowledge.

## 4. Materials and Methods

### 4.1. Cases Report

Family 1

**Figure 4:**
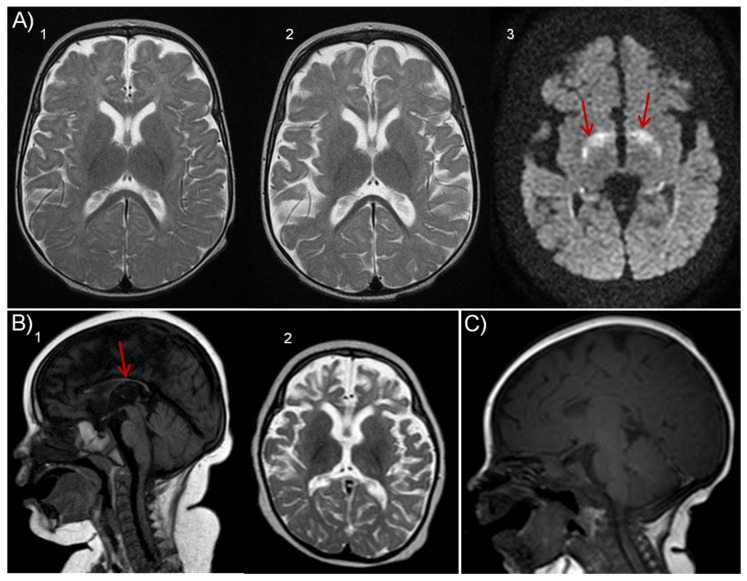
**Brain imaging**. (**A**) Patient 1.2: T2 axial MR images show cortical atrophy evolution from the initial MR (1) and after 3 months evolution (2); (3) diffusion-weighted image shows increased paleo-lenticular signal intensity. (**B**) Patient 1.1: Sagital T1 demonstrate corpus callosum thinning (1) and diffuse cortical and subcortical atrophy in axial T2 (2). (**C**): Patient 2.1 Sagital T1 image depicts corpus callosum thinning.

Patient 1.2 (P1.2). Index case. The patient was the third daughter of healthy, third-degree, consanguineous gypsy parents. She had two older siblings, one of them was healthy, while the other died at 7.5 months of age (P1.1) with clinical symptoms very close to that of the patient we report here. Pregnancy was uneventful; delivery was at term, but urgent cesarean section was needed due to loss of fetal well-being. Apgar score was 8/1. She was admitted to the Neonatal Intensive Care Unit (NICU), where she remained for several weeks. During this period, the main problems were moderate laryngomalacia, central apnea, the development of hypertrophic cardiomyopathy, and several septic pictures, which caused intestinal ischemia requiring extensive intestinal resection. At 4 months of age, the patient presented a severe episode of non-ketotic hypoglycemia without insulin, C peptide, or cortisol alterations. At 6 months old, repeated asymptomatic non-ketotic hypoglycemia was detected. At that age, the clinical picture showed severe microcephaly, global hypotonia, and feeding difficulties that required percutaneous gastrostomy. Plasma and urine metabolite analyses aimed at discarding inherited metabolic diseases, including very long-chain fatty acids (VLCFA), acylcarnitines, and isoforms of sialotransferrin, organic acids, and oligosaccharides, were all normal. Mitochondrial respiratory chain activities in muscle and deuterated palmitate flux in fibroblasts were also unaltered. Plasma copper and ceruloplasmin were normal. Karyotype showed no abnormalities. At 2 years old, she began to experience focal motor epileptic seizures with bilateral tonic evolution. Antiepileptic drugs were administered, to which she was refractory. Serial electroencephalogram (EEG) showed abundant multifocal epileptiform activity. Brain magnetic resonance imaging (MRI) showed progressive cerebral atrophy and gliosis of the temporal lobes with symmetrical bilateral affectation and marked atrophy of the corpus callosum (Figure 4A). Ophthalmological evaluation and MRI of the hypophysis were normal.

The clinical course was characterized by asymptomatic non-ketotic hypoglycemia, short stature, undetectable levels of somatomedin C, and insulin-like growth factor-binding protein 3 (IGFBP3). She evolved with favorable resolution of the cardiomyopathy with minimal hypertrophy of the ventricular septum. Neurologically, she continued to have severe global hypotonia, progressive microcephaly, and neurodevelopmental arrest. Seizures remained stable despite refractoriness. The patient died at the age of 4 years in the context of a hypoxemic respiratory infection.

Patient 1.1 (P1.1). She was studied retrospectively because of the diagnosis of her young sister (P1.2). Pregnancy was uneventful. Delivery was induced at 36 weeks of gestation due to intrauterine growth retardation. Birth weight and Apgar score were 2310 g and 5/8, respectively. She was admitted to the NICU due to respiratory distress and hypotonia. Hypoglycemia was detected, and intravenous administration of glucose, as well as a nasogastric tube feeding during the first 4 days of life, were required. It is of note the detection of apnea pauses were responsive to stimulation and oxygen supplement. She was discharged at 10 days of life but was re-admitted at 1-month-old due to gastroenteritis (rotavirus and adenovirus positive). Physical examination revealed global hypotonia, weight, and height delay. Despite the improvement of the infectious picture, irritability, vomiting, and apnea pauses persisted, as well as non-ketotic hypoglycemia. A nasogastric tube was placed again for continuous night feeding. She started to present oral automatisms and epileptic seizures that subsided with Levetiracepam. She was discharged 40 days after admission with anti-GERD therapy, as well as home oxygen therapy, and carnitine and riboflavin supplementation due to suspicion of mitochondrial disease.

Biochemical studies showed high plasma lactate 8 mmol/L (R.V.: <2), persistent non-ketotic hypoglycemia, and slight hypertransaminasemia. VLCFA, acylcarnitines, plasma sialotransferrin isoforms, and oligosaccharides were normal. Organic acids in urine showed a slight increase in lactate. Mitochondrial respiratory chain activities in muscle were normal. Endocrinology investigations showed low insulin-like growth factor I (IGF1) 24 ng/mL (C.V.: 55-327), while insulin/glucose ratio, adrenocorticotropic hormone (ACTH), growth hormone (GH), and IGFBP3 were normal. Abdominal and cardiac ultrasound were also normal. Brain MRI showed thinned corpus callosum (Figure 4B).

She was admitted again at 4 months of age due to bronchiolitis and pneumonia with severe apnea pauses. At 7 months of age, microcephaly, global hypotonia, and delayed psychomotor development were the most relevant clinical signs. The patient died at home at 7.5 months of age due to cardiorespiratory arrest.

Family 2 (Patient 2.1)

Patient 2.1 (P2.1). Index case. She was the second daughter of healthy, non-consanguineous gypsy parents. She has a healthy 5-year-old brother. Pregnancy and delivery were uneventful. Height, weight, and head circumference were normal. Apgar score was 8/9. She was admitted to the neonatal unit due to hypoglycemia, hypotonia, and hypoxemia with apnea pauses. She needed a nasogastric tube and oxygen support. She was discharged at 40 days of age.

During intercurrent infectious processes, she required several admissions due to exacerbation of the symptoms. At 4 months of age, she presented acute gastroenteritis with dehydration and metabolic acidosis (without hypoglycemia). At 6 months old, episodes suggestive of seizures appeared, initially with spasms in extension and normal EEGs but later confirmed by video-EEG. The episodes were initially treated with Levetiracetam without achieving any response but subsided with valproic acid. Clonacepam was added to the treatment due to marked irritability. At 6.5 months old, she was admitted again due to fever and hypoglycemia. At 8 months, coinciding with a MERS coronavirus infection, respiratory symptoms worsened, and preprandial non-ketotic hypoglycemia stood out.

Metabolite studies in plasma and urine showed normal amino acid, organic acid, and acylcarnitine profiles, as well as normal sialotransferrin isoforms. Lactate and neurotransmitters in cerebrospinal fluid were also normal. Endocrinology studies showed non-ketotic hypoglycemia with normal ACTH but low insulin 0.4 µUI/mL (C.V.: 0.84–31.1), cortisol 1.6 g/dL (C.V.: 2.6–23), IGF1 < 15 ng/mL (C.V.: 55–327) and IGFBP3 < 0.5 µg/mL (C.V.: 0.7–3.6). Fecal elastase was also low, 181µg/g (C.V.: >200)

Abdominal ultrasound and echocardiography were normal. Brain MRI at 1 month of age was normal, but at 4 months old, the corpus callosum was thinned, showing a certain degree of atrophy, and on examination at 7 months old, a greater degree of atrophy, as well as hypomyelination and alteration of the basal ganglia were detected (Figure 4C).

In summary, clinical evolution was characterized by global hypotonia, progressive microcephaly, and psychomotor development arrest. She never acquired head support nor the ability to roll over or sit. Likewise, she presented symptoms suggestive of gastroesophageal reflux, with apnea pauses and digestive problems. She died at home when she was 10 months old; she presented an apnea pause that caused her death.

### 4.2. Whole Exome Sequencing (WES)

WES was performed at the Centre Nacional d’Anàlisi Genòmica (CNAG-CRG, Barcelona, Spain). For exome enrichment, we used the Nimblegen SeqCap EZ MedExome+mtDNA 47Mb capture kit, followed by sequencing using the Illumina HiSeq 2000 genome analyzer platform. The primary data files (FASTQ files) were analyzed using the pipeline developed by CNAG-CRG [16]. Sequence reads were mapped to the Human genome build hg19/GRCh37. The variant calls were analyzed using the URDCAT genome-phenome analysis platform (https://rdcat.cnag.crg.eu/, accessed on 14 January 2022), filtered by frequency (allele frequency < 1% in population databases, including 1000G and gnomAD), and by the functional impact on the encoded protein, as well as for the clinical and biochemical phenotype of the patient.

### 4.3. Protein Expression Analysis

Western blot of CCDC186 protein was performed in muscle biopsy of P1.1 and in fibroblasts of P1.2, as well as control tissues. Material from P2.1 was not available. Muscle extracts were prepared in tissue extraction buffer (250 mM mannitol, 75 mM sucrose, 10 nM Tris-HCl, 0.1 mM EDTA) and centrifuged at 600 g at 4 °C for 20 min. Fibroblasts were lysed using RIPA buffer containing a protease inhibitor cocktail (1862209, Merck, Darmstadt, Germany). Briefly, cells were scraped in RIPA lysis buffer, incubated on ice for 10 min, and centrifuged at 10,000 g at 4 °C for 10 min. Protein extracts (40 ug) were subjected to SDS-PAGE, electroblotted, and visualized by immunostaining with specific antibodies followed by colorimetric detection (Opti-4CNTM Substrate Kit, Bio-Rad, Hercules, CA, USA). The antibodies used in this study were: anti-CCDC186 (HPA018019, Sigma Aldrich, Burlington, MA, USA) and anti-beta actin (ab115777, Abcam, UK). Membrane images were quantified using ImageJ software 1.53t.

### 4.4. RNA Sequencing

RNA-seq was also performed at CNAG-CRG. The quality control of the total RNA was done using the Qubit^®^ RNA HS Assay (Life Technologies, Carlsbad, CA, USA) and RNA 6000 Nano Assay on a Bioanalyzer 2100 (Agilent, Santa Clara, CA, USA). Libraries were prepared using the TruSeq^®^ Stranded mRNA LT Sample Prep Kit (Illumina Inc., San Diego, CA, USA, Rev.E, October 2013). The libraries were sequenced on a HiSeq 2500 (Illumina) in paired-end mode (2 × 76 bp). Primary data analysis, image analysis, base calling, and quality scoring of the run were processed using the manufacturer’s software Real Time Analysis (RTA 1.18.66.3), followed by the generation of FASTQ sequence files. Reads from RNA-seq were demultiplexed and then mapped with STAR (v2.7.0a) to the hg19 genome assembly. Analysis of the aligned data was done using the “Detection of RNAseq Outliers pipeline” (DROP) [17,18,19] in order to detect aberrantly expressed genes, altered splicing events, and monoallelic expression (MAE) of rare variants. As controls, a cohort of 313 fibroblasts from patients with Mendelian disorders [20] was used. RNA-seq normalized counts are provided in the Supplementary Materials, Tables S1–S3.

### 4.5. Functional Annotation of Differentially Expressed Genes

Statistical over-representation test was performed on the significantly (OUTRIDER’s *p* < 0.05) down and upregulated genes (N = 368 and 384, respectively) using PANTHER Classification System (http://www.pantherdb.org/, accessed on 14 March 2023). Only PANTHER Gene Ontology (GO)-Slim Biological Processes were analyzed.

### 4.6. Statistics and Data Analysis

Statistical analyses were performed using the two-tailed Student’s *t*-test to compare the means of two independent groups of normally distributed data. Data were reported as the mean ± SD. *p*-values lower than 0.05 were considered statistically significant. The RNA-seq dataset was analyzed with the R Statistical Computing environment (https://www.r-project.org/, accessed on 14 March 2023) for graphic generation.

## 5. Conclusions

In summary, our study provides the first functional evidence that mutations in *CCDC186* have a pathogenic effect on the encoded protein and reinforces *CCDC186* as a new disease-associated gene. In addition, mutations in *CCDC186* could explain the combined endocrine and neurologic alterations detected in the patients.

## Figures and Tables

**Table 1:** Main clinical and molecular aspects of a previously reported CCDC186 patient compared with the patients reported in the present study. The patient reported by Monies et al. [14] was not included in the table due to the incomplete clinical description.

		Brugger et al. [15]	This Report		
			P1.1	P1.2	P2.1
**Age at onset**		4 months	Neonatal	Neonatal	Neonatal
**Exitus**		Unknown. Age at last follow-up: 15 months	7 months	4 years	10 months
**Origin**		Senegalese	Spanish gypsy	Spanish gypsy	Spanish gypsy
**Consanguinity**		Yes	Yes	Yes	No
**Congenital malformations**		Infundibular pulmonary stenosis	No	Laryngomalacia	No
**Growth**	**IUGR**	Yes	Yes	No	No
	**Failure to thrive**	Yes	Yes	Yes	Yes
	**Microcephaly**	Yes	Yes	Yes (severe)	Yes (severe)
**Development**	**Developmental delay**	Yes (severe)	Yes (severe)	Yes	Yes (severe)
**Neurological findings**	**Muscular hypotonia**	Yes	Yes	Yes	Yes
	**Refractory seizures**	Yes	Yes	Yes	Yes
	**MRI findings**	Yes Frontotemporal atrophy	Yes Thin corpus callosum	Yes Thin corpus callosum Progressive frontotemporal atrophy	Yes Thin corpus callosum Progressive atrophy Hypomyelination
**Heart findings**		NR	No	Yes Hypertrophic cardiomyopathy	No
**Gastrointestinal findings**		Yes Vomiting, Swallowing Obstipation Exocrine pancreas insufficiency	Yes Gastroesophageal reflux	Yes Intestinal ischemia	Yes Gastroesophageal reflux Swallowing Exocrine pancreas insufficiency
**Endocrinologic findings**		Yes Hypothyroidism, Suspected endocrine pancreas insufficiency	Yes Non-ketotic hypoglycemia, low IGF1	Yes GH deficiency, non-ketotic hypoglycemia	Yes Non-ketotic hypoglycemia, low Insulin, cortisol, IGF1, BP3IGFl, and Elastase
**Respiratory findings**		NR	Apneas	Apneas	Apneas
**Ophthalmologic findings**		Lack of fixation	Lack of fixation	No	Lack of fixation
**Auditory findings**		Hyperacusia, left side	No	No	No
**Genotype**		c. [767C>G]; [767C>G]	c. [2215C>T]; [2215C>T]	c. [2215C>T]; [2215C>T]	c. [2215C>T]; [2215C>T]
**Effect on protein**		p. [Ser256Ter]; [Ser256Ter]	p. [Arg739Ter]; [Arg739Ter]	p. [Arg739Ter]; [Arg739Ter]	p. [Arg739Ter]; [Arg739Ter]

## Data Availability

Data supporting reported results is available as Supplementary Materials. Additional data that support the findings of this study are available from the corresponding authors, upon reasonable request.

## Supplementary Materials

The following supporting information can be downloaded at: https://www.mdpi.com/article/10.3390/ijms241512319/s1.
